# Gastroschisis prevalence patterns in 27 surveillance programs from 24 countries, International Clearinghouse for Birth Defects Surveillance and Research, 1980–2017

**DOI:** 10.1002/bdr2.2306

**Published:** 2024-02

**Authors:** Marcia L. Feldkamp, Mark A. Canfield, Sergey Krikov, David Prieto-Merino, Antonin Šípek, Nathalie LeLong, Emmanuelle Amar, Anke Rissmann, Melinda Csaky-Szunyogh, Giovanna Tagliabue, Anna Pierini, Miriam Gatt, Jorieke E. H. Bergman, Elena Szabova, Eva Bermejo-Sánchez, David Tucker, Saeed Dastgiri, María Paz Bidondo, Aurora Canessa, Ignacio Zarante, Paula Hurtado-Villa, Laura Martinez, Osvaldo M. Mutchinick, Jorge Lopez Camelo, Adriana Benavides-Lara, Mary Ann Thomas, Shiliang Liu, Wendy N. Nembhard, Elizabeth B. Gray, Amy E. Nance, Pierpaolo Mastroiacovo, Lorenzo D. Botto

**Affiliations:** 1 Division of Medical Genetics, Department of Pediatrics, University of Utah School of Medicine, Salt Lake City, Utah, USA; 2 Birth Defects Epidemiology and Surveillance Branch, Texas Department of State Health Services, Austin, Texas, USA; 3 Faculty of Medicine, Universidad de Alcalá, Madrid, Spain; 4 Czech Republic Department of Medical Genetics, Thomayer Hospital, Prague, Czech Republic; 5 Université Paris Cité, Centre of Research in Epidemiology and StatisticS (CRESS), Obstetrical Perinatal and Pediatric Epidemiology Research Team (EPOPé), INSERM, INRA, Paris, France; 6 France REMERA, Registre des malformations en Rhône Alpes, Hospices Civils de Lyon, Lyon, France; 7 Malformation Monitoring Centre Saxony-Anhalt, Medical Faculty Otto-von-Guericke-University Magdeburg, Magdeburg, Germany; 8 Hungarian Congenital Anomalies Registry and Rare Diseases Centre, National Center for Public Health and Pharmacy, Budapest, Hungary; 9 Lombardy Congenital Anomalies Registry, Cancer Registry Unit, Fondazione IRCCS, Istituto Nazionale dei tumori, Milan, Italy; 10 Unit of Epidemiology of Rare Diseases and Congenital Anomalies, Institute of Clinical Physiology, National Research Council and Fondazione Toscana Gabriele Monasterio, Tuscany Registry of Congenital Defects, Pisa, Italy; 11 Malta Congenital Anomalies Registry, Directorate for Health Information and Research, Pieta, Malta; 12 Department of Genetics, University of Groningen, University Medical Center Groningen, Groningen, the Netherlands; 13 Faculty of Public Health, Slovak Medical University in Bratislava, Bratislava, Slovak Republic; 14 ECEMC (Spanish Collaborative Study of Congenital Malformations), CIAC (Research Center on Congenital Anomalies), Institute of Rare Diseases Research (IIER), Instituto de Salud Carlos III, Madrid, Spain; 15 Congenital Anomaly Register & Information Service for Wales, Public Health Wales, Knowledge Directorate, Singleton Hospital, Sketty Lane, Swansea, UK; 16 Health Services Management Research Centre, Tabriz University of Medical Sciences, Tabriz, Iran; 17 National Network of Congenital Anomalies of Argentina (RENAC), National Institute of Epidemiology (INE), National Administration of Laboratories and Health Institutes, National Ministry of Health Institutes, Buenos Aires, Argentina; 18 Regional Register Congenital Malformation Maule Health Service (RRMC-SSM), Maule, Chile; 19 Instituto de Genética Humana, Pontificia Universidad Javeriana Bogotá, Bogotá, Colombia; 20 Facultad de Ciencias de la Salud, Pontificia Universidad Javeriana Cali, Cali, Colombia; 21 Mexico ReDeCo, Monterrey, Nuevo Leon, Mexico; 22 Department of Genetics, Instituto Nacional de Ciencias Médicas y Nutrición Salvador Zubirán, RYVEMCE, Registry and Epidemiological Surveillance of Congenital Malformations, Mexico City, Mexico; 23 ECLAMC, Center for Medical Education and Clinical Research (CEMIC-CONICET), Buenos Aires, Argentina; 24 Costa Rican Birth Defects Register Center (CREC), Costa Rican Institute for Research and Teaching in Nutrition and Health (INCIENSA), Cartago, Costa Rica; 25 Department of Medical Genetics and Pediatrics, Alberta Congenital Anomalies Surveillance System, Alberta Children’s Hospital, Calgary, Alberta, Canada; 26 Canadian Congenital Anomalies Surveillance System (CCASS), Centre for Surveillance and Applied Research, Public Health Agency of Canada, Ottawa, Ontario, Canada; 27 Department of Epidemiology, Fay W. Boozman College of Public Health, University of Arkansas for Medical Sciences and Arkansas Reproductive Health Monitoring System, Arkansas Children’s Research Institute, Little Rock, Arkansas, USA; 28 Metropolitan Atlanta Congenital Defects Program, National Center on Birth Defects and Developmental Disabilities, Centers for Disease Control and Prevention, Atlanta, Georgia, USA; 29 Utah Birth Defect Network, Office of Children with Special Care Needs, Division of Family Health, Utah Department of Health and Human Services, Salt Lake City, Utah, USA; 30 International Center on Birth Defects, International Clearinghouse for Birth Defects Surveillance and Research, Rome, Italy; 31 Department of Pediatrics, The University of Utah, Salt Lake City, Utah, USA

**Keywords:** gastroschisis, geographic region, multinational, prevalence, surveillance

## Abstract

**Background:**

Gastroschisis is a serious birth defect with midgut prolapse into the amniotic cavity. The objectives of this study were to evaluate the prevalence and time trends of gastroschisis among programs in the International Clearinghouse for Birth Defects Surveillance and Research (ICBDSR), focusing on regional variations and maternal age changes in the population.

**Methods:**

We analyzed data on births from 1980 to 2017 from 27 ICBDSR member programs, representing 24 countries and three regions (Europe^+ (includes Iran)^, Latin America, North America). Cases were identified using diagnostic codes (i.e., 756.7, 756.71, or Q79.3). We excluded cases of amniotic band syndrome, limb–body wall defect, and ruptured omphalocele. Programs provided annual counts for gastroschisis cases (live births, stillbirths, and legally permitted pregnancy terminations for fetal anomalies) and source population (live births, stillbirths), by maternal age.

**Results:**

Overall, gastroschisis occurred in 1 of every 3268 births (3.06 per 10,000 births; 95% confidence intervals [CI]: 3.01, 3.11), with marked regional variation. European^+^ prevalence was 1.49 (95%CI: 1.44, 1.55), Latin American 3.80 (95%CI: 3.69, 3.92) and North American 4.32 (95%CI: 4.22, 4.42). A statistically significant increasing time trend was observed among six European^+^, four Latin American, and four North American programs. Women <20 years of age had the highest prevalence in all programs except the Slovak Republic.

**Conclusions:**

Gastroschisis prevalence increased over time in 61% of participating programs, and the highest increase in prevalence was observed among the youngest women. Additional inquiry will help to assess the impact of the changing maternal age proportions in the birth population on gastroschisis prevalence.

## 1 | INTRODUCTION

Gastroschisis is an abdominal defect that results in either a separation of the amnio-ectodermal junction or split in the base of the umbilical cord permitting the midgut to prolapse into the amniotic cavity (Bargy & Beaudoin, 2014; Beaudoin, 2018; Rittler et al., 2013). Based on the human evidence, this midgut prolapse occurs on the thin upper-right side (pars flaccida) of the umbilical ring during the normal physiologic hernia in the first trimester (Bargy & Beaudoin, 2014; Beaudoin, 2018; Rittler et al., 2013; Shaw, 1975), whereas the abdominal wall per se (comprised of skin, muscle, etc.) remains essentially intact. What factor(s) negatively impact the connection at the amnio-ectodermal junction is unknown. At birth, gastroschisis presents with the midgut prolapsed outside the abdomen through an aperture, most often to the right of the umbilicus (the umbilicus remains attached only on the left side). The prolapsed organs are not covered by a membrane. However, at times the defect’s presentation requires careful differentiation from a ruptured omphalocele, another birth defect with a different origin, risk factors, and epidemiologic patterns. Distinctive features of omphalocele are an enlarged umbilical ring with the umbilical cord centrally inserted into the covering membrane of the prolapsed organs, or rarely rupture (Khan et al., 2019).

Unlike other major birth defects, gastroschisis prevalence has increased over the past several decades (Jones et al., 2016), and this pattern has raised questions and concerns. During the 1960s and 1970s, the baseline prevalence of gastroschisis was estimated at approximately 1 in every 50,000 births. Lindham (1981) first reported a 30% increase in gastroschisis prevalence between 1965 and 1976 in Sweden. This increase in prevalence was then noted by others (Barboza-Argüello & Benavides-Lara, 2018; Calzolari et al., 1993; Goldbaum et al., 1990; Hemminki et al., 1982; Kirby et al., 2013; Loane et al., 2007; Martinez-Frias et al., 1984; Roeper et al., 1987; Tan et al., 1996), including by Castilla et al. (2008) who reported a 10 to 20-fold increase in the prevalence of gastroschisis in several countries over time since the 1960s.

A second unusual finding in gastroschisis is the maternal age pattern, namely, the consistent finding that regardless of overall prevalence, the highest prevalence of gastroschisis within a population is observed among women <20 years of age (Castilla et al., 2008; Kirby et al., 2013; Salinas-Torres et al., 2018).

These two findings, time trends and maternal age risk, raise the question of whether the increasing prevalence is present in all age groups or only those at highest risk (i.e., women <20 years of age). In general, the observed increase in prevalence was reported primarily among women <20 years (Barboza-Argüello & Benavides-Lara, 2018; Bermejo et al., 2006; Kilby, 2006; Kirby et al., 2013; Laughon et al., 2003; Reid et al., 2003) whereas others have reported a decrease in this age group (Calderon et al., 2019; Loane et al., 2007). Some investigators have reported an increase in prevalence in all maternal age categories (Jones et al., 2016; Kazaura et al., 2004; Salinas-Torres et al., 2018; Vo & Langlois, 2015). The increase in prevalence during the past several decades and the highest prevalence among women under 20 years of age are unique to gastroschisis (Bhat et al., 2020; Stallings et al., 2019).

A third finding relates to striking differences in prevalence in different regions and countries (Castilla et al., 2008). Cluster investigations have been conducted on gastroschisis without identifying specific new risk factors (Materna-Kiryluk et al., 2016; Yazdy et al., 2015). Loane et al. (2007) reported gastroschisis prevalence among 25 registries from 15 European countries during 1980–2002 ranging between 0.31 (Italy-Tuscany) and 4.48 (Germany-Mainz) per 10,000 births. Mastroiacovo et al. (2006) analyzed data from 25 registries included in the International Clearinghouse for Birth Defects Surveillance and Research (ICBDSR) and reported a statistically significant increasing trend of gastroschisis prior to 2004 in 14 (56%) registries compared to no increasing trend for 36 other types of birth defects. Though not a focus of this study, the reasons for such disparities in prevalence are not known but perhaps behaviors and exposures to environmental factors differ in different areas of the world.

This study updates previously published data on gastroschisis prevalence among multiple countries (Loane et al., 2007; Mastroiacovo et al., 2006) with three main objectives: (1) evaluate the multinational prevalence and trends of gastroschisis between 1980 and 2017 among participating ICBDSR member programs; (2) compare prevalence in three geographic regions, by surveillance program and maternal-age groups; and (3) evaluate population changes in the proportion of births by maternal age groups during the study period.

## 2 | METHODS

The ICBDSR is an international voluntary and non-profit organization in official relations with the World Health Organization (WHO) whose members conduct birth defect surveillance in their country, region, or at the state-level (Mastroiacovo et al., 2007). Created in 1974, its mission is to use the power of international public health monitoring to track, study, and ultimately prevent and improve outcomes related to birth defects (Botto et al., 2006).

Programs were eligible to participate if they could provide relevant data on gastroschisis cases among all pregnancy outcomes (live births, stillbirths, and elective terminations of pregnancy for fetal anomaly [ETOPFA] if legally permitted) as well as population births (live births, stillbirths) by birth year and maternal age at delivery/termination.

The following criteria were used for case inclusion: all pregnancy outcomes (live births, stillbirths, and ETOPFA) were included that listed gastroschisis (International Classification of Diseases, Ninth Revision [ICD-9] code *756.7*, British Pediatric Association [BPA] modified code *756.71*, and International Classification of Diseases, 10th Revision [ICD-10] code *Q79.3*) during the program’s surveillance time period. Cases considered or listed with a limb–body wall defect, an abdominal defect due to amniotic bands, omphalocele, or a ruptured omphalocele were excluded.

Of the 43 ICBDSR members, 27 programs participated in this study representing a total of 24 countries. Surveillance programs were divided into three regions based on geography. European^+^ region included programs from the Czech Republic, France (Rhone-Alpes and Paris), Germany Saxony-Anhalt, Hungary, Italy (Lombardy and Tuscany), Malta, Northern Netherlands, Slovak Republic, Spain, Wales, *plus* (^+^) Iran. The Latin American region included programs from South America (Argentina, Chile, Colombia-Bogota, Colombia-Cali), Central America (Costa Rica) and North America (Mexico Nuevo León, Mexico’s Registry and Epidemiological Surveillance of Congenital Malformations, Department of Genetics, Instituto Nacional de Ciencias Médicas y Nutrición Salvador Zubirán, Mexico City [RYVEMCE]), and the Latin American Collaborative Study of Congenital Malformations (ECLAMC) a hospital-based program with data from nine countries in South America (Argentina, Bolivia, Brazil, Chile, Colombia, Ecuador, Paraguay, Peru, and Venezuela). The North American region included programs from Canada (Alberta and nation-wide) and the United States (Arkansas, Atlanta, Texas, and Utah). Surveillance characteristics are shown for each participating program (Table 1). Hospital-based surveillance systems included two European^+^ programs (Spain ECEMC and Iran TRoCA) and all Latin America programs (with the exception of Costa Rica). The remaining programs were population-based. Among the 27 participating programs, 22 were able to provide counts by maternal age and year of birth for cases and the source population. Five programs (Hungary, Iran, Colombia-Bogota, Colombia-Cali, and South America ECLAMC) were either unable to provide complete counts by birth year and maternal age for the denominator-source population or maternal age was missing for many of the case and/or population counts to obtain accurate estimates. Because only four programs were able to submit surveillance data prior to 1980, all analyses for prevalence and maternal-age specific prevalence were calculated for the period between 1980 and 2017. As shown in Table 1, some programs only began monitoring gastroschisis during the 1990’s.

### 2.1 | Statistical analysis

We calculated prevalence using the following formula: total gastroschisis cases (among stillbirths + live births + ETOPFA)/total births (stillbirths + live births from the source population). Prevalence was expressed as cases per 10,000 births. 95% confidence intervals (CI) were calculated using Poisson approximation of the binomial distribution when cell sizes were five or greater and exact binominal 95% CIs for cell sizes <5. For each region and program within a region, we calculated prevalence for each decade the program contributed data and for each maternal age category (<20, 20–24, 25–29, and ≥30 years of age). To assess trends over time for overall prevalence and by maternal age categories, we used the Cochrane-Armitage test for categorical data and the Jonckheere-Terpstra test for distribution-free non-parametric analysis of an independent variable that is either continuous or order dependent (Jonckheere, 1954; Terpstra, 1952). For each region, we used a three-year moving average (smoothing technique) to plot maternal age-specific prevalence by year. We used two-sided tests to evaluate statistical significance at a p value<0.05. All analyses were conducted with SAS software 9.4 (SAS Institute Inc., Cary, NC, 2013) or R software 4.10 (R Core Team, 2020).

## 3 | RESULTS

The study population included 14,020 gastroschisis cases (live births, stillbirths, and ETOPFA) among 45,755,137 total births (stillbirths + live births), from 27 surveillance programs representing 24 countries and three regions during birth years 1980–2017 (Table 2).

### 3.1 | Prevalence

**FIGURE 1:**
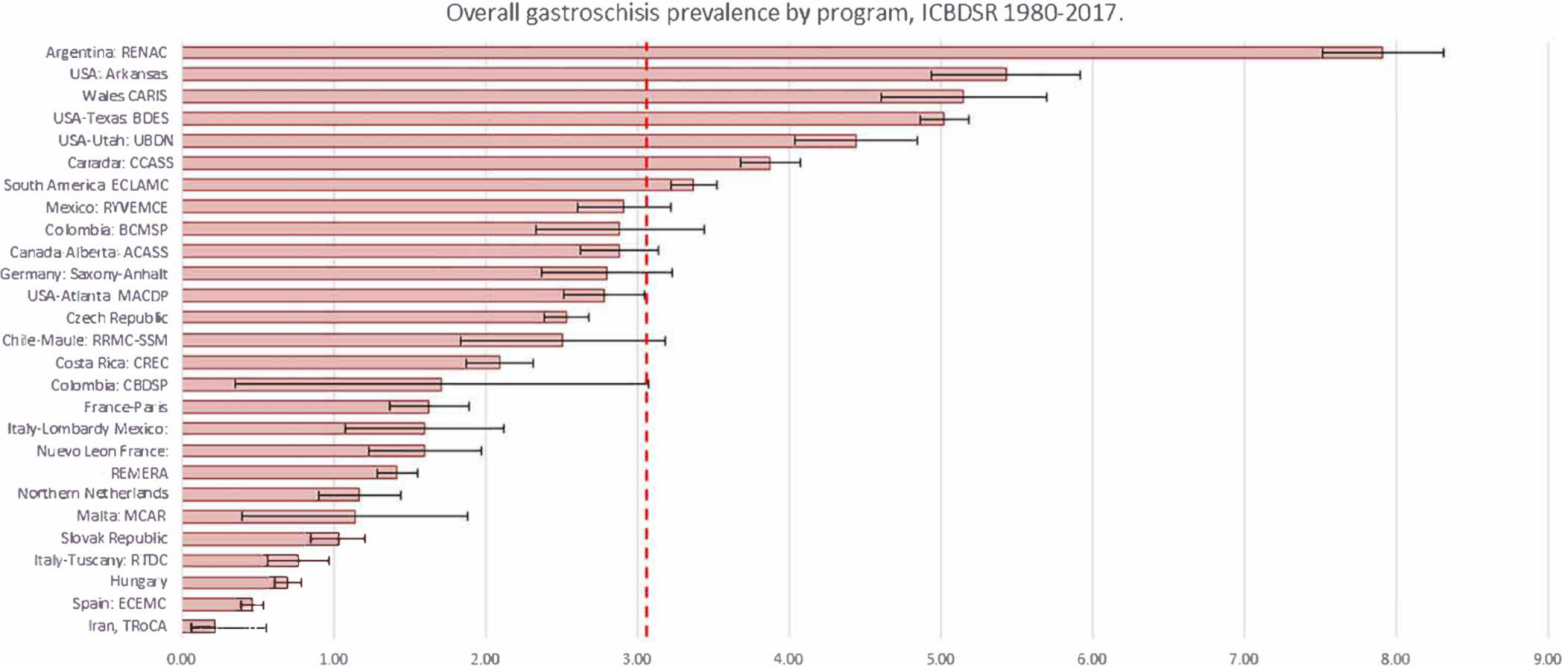
Prevalence (95%CI) of gastroschisis by program, International Clearinghouse for Birth Defects Surveillance and Research (ICBDSR), 1980–2017. Red dotted line represents overall average prevalence 3.06 (95% confidence interval: 3.01, 3.11) per 10,000 births. CARIS, Congenital Anomaly Register and Information Services for Wales; CCASS, Canadian Congenital Anomalies Surveillance System; CREC, Costa Rican Birth Defects Register Center; ECLAMC, Latin American Collaborative Study of Congenital Malformations; ECEMC, Spanish Collaborative Study of Congenital Malformations; MCAR, Malta Congenital Anomalies Register; REMERA, Registre des Malformations en Rhône-Alpes; RENAC, National Network of Congenital Anomalies of Argentina; RRMC-SSM, Registro de malformaciones congénitas del Servicio de Salud Maule; RTDC, Registro Toscano Difetti Congeniti; RYVEMCE, Registration and Epidemiologic Surveillance of Congenital Malformations; TRoCA, Tabriz Registry of Congenital Anomalies.

Gastroschisis case counts, total birth population counts (live births and stillbirths), and overall prevalence per 10,000 births are shown for each geographic region and surveillance program within the region (Table 2). Overall, gastroschisis prevalence occurred in one of every 3268 births (3.06 per 10,000 births; 95% CI: 3.01, 3.11). Gastroschisis prevalence was lower in the European^+^ region compared to the prevalence observed in both Latin America and North America. Among the 23 programs with sufficient data, decade-specific prevalence demonstrated a statistically significant increasing trend among 14 programs (61%), a significantly decreasing trend in one program (4%), and no change in prevalence over time in eight programs (35%) (Table 2). Four programs did not have data before 2010 to calculate time trends by decade. Seven programs had prevalence estimates above the overall average of 3.06 per 10,000 births for all programs (Figure 1).

### 3.2 | Maternal age

Table 3 summarizes prevalence estimates for maternal age groups within each region and program within the region. The region-specific prevalence was lower in the European^+^ region for each maternal age group, compared to both Latin America and North America. Except for the Slovak Republic, women <20 years of age had the highest prevalence and as maternal age increased, maternal-age specific gastroschisis prevalence decreased.

**FIGURE 2:**
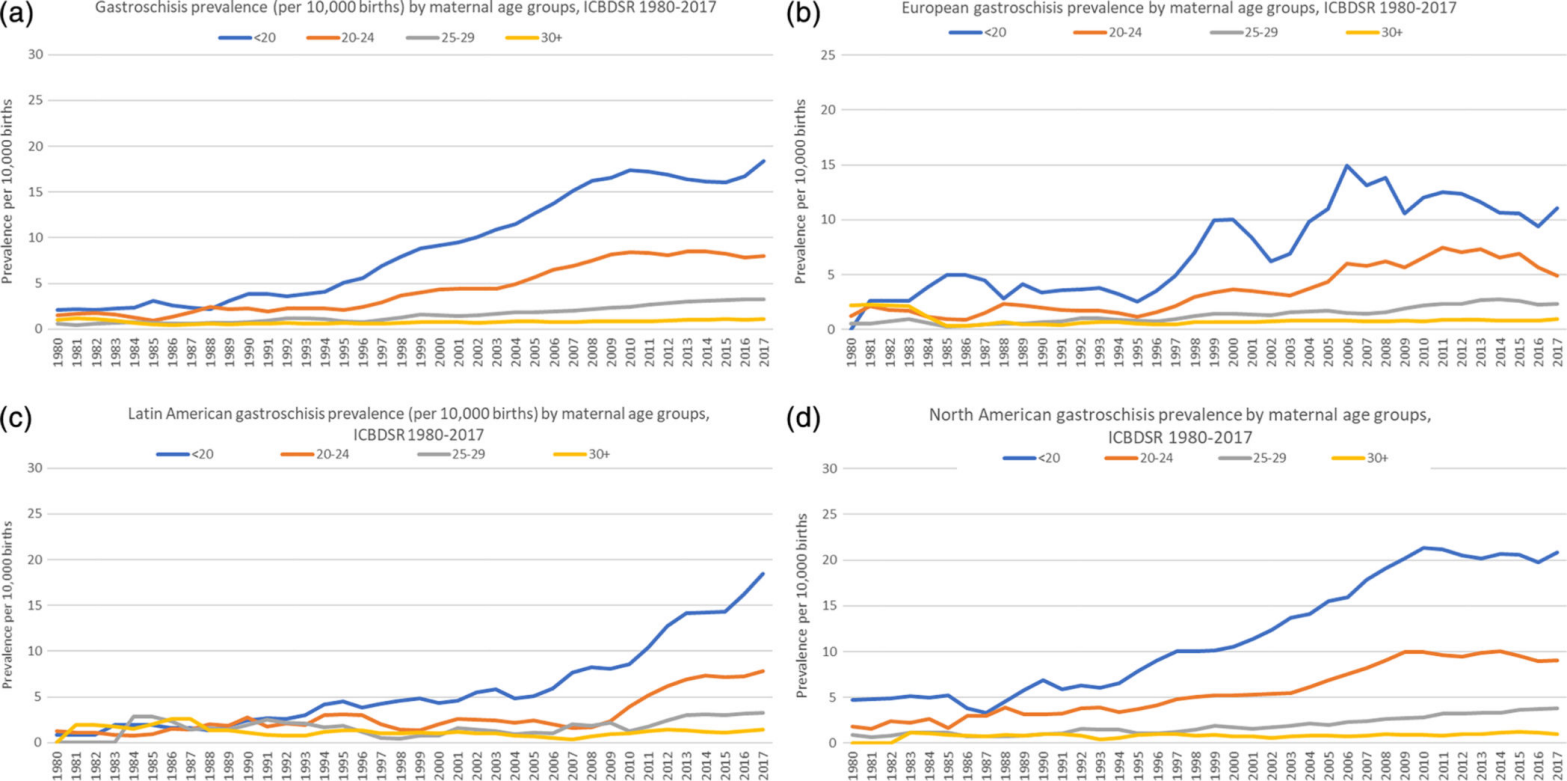
(a) Overall three-year average smoothed prevalence for gastroschisis by maternal age groups for 22 birth defect programs representing 15 countries, International Clearinghouse for Birth Defects Surveillance and Research (ICBDSR), 1980–2017. Refer to Table 1 for years included for maternal age by program. (b) European three-year moving average of prevalence for gastroschisis by maternal age groups for 11 birth defect programs representing 9 countries, International Clearinghouse for Birth Defects Surveillance and Research (ICBDSR), 1980–2017. Refer to Table 1 for years included for maternal age by program. (c) Latin America three-year moving average of prevalence for gastroschisis by maternal age groups for five birth defect programs representing 11* countries, International Clearinghouse for Birth Defects Surveillance and Research (ICBDSR), 1980–2017. Refer to Table 1 for years included for maternal age by program. *Mexico and nine countries represented from South America. (d) North America three-year moving average of prevalence for gastroschisis by maternal age groups for six birth defect programs representing two countries, International Clearinghouse for Birth Defects Surveillance and Research (ICBDSR), 1980–2017. Refer to Table 1 for years included for maternal age by program.

The three-year moving average stratified by maternal age, overall prevalence and by region, are shown in Figure 2a–d. For each maternal age group, a statistically significant increasing time trend was observed overall (Figure 2a), for European^+^ (Figure 2b), Latin America (Figure 2c), and North America (Figure 2d) programs (detailed trend data not shown).

### 3.3 | Birth population by maternal age

We evaluated each of the 22 program’s maternal age-specific proportion of births over time to determine if changes occurred since 1980. We observed a statistically significant decrease over time in the proportion of births among women under 20 years of age in 17 (77%) programs (nine European, two Latin American, six North American), among women 20–24 years of age in 16 (73%) programs (eight European, two Latin American, six North American), and among women 25–29 years of age in 10 (45%) programs (seven European, three North American). In comparison, 18 (82%) programs had an increase in the proportion of births among women ≥30 years of age (nine European, one Latin American, six North American) (data not shown).

## 4 | DISCUSSION

In this international study from 27 programs in 24 countries, we observed three main findings. First, 14 (61%) programs with sufficient data representing 18 countries (six European^+^, 10 Latin American which includes nine from the ECLAMC program, and two North American) showed a significant increase in gastroschisis prevalence over time, nine of which were based on data from 1980 to 2017. Second, prevalence of gastroschisis among programs in Latin America and North America was more than two times that observed in European^+^ programs. Third, in all programs, with one exception, the prevalence was highest among women under 20 years of age, with progressively lower prevalence as maternal age increased.

Our findings are consistent with prior observations of increasing time trends (Barboza-Argüello & Benavides-Lara, 2018; Calderon et al., 2019; Jones et al., 2016; Kirby et al., 2013; Loane et al., 2007; Salinas-Torres et al., 2018) and an inverse relationship with maternal age (Barboza-Argüello & Benavides-Lara, 2018; Bourque et al., 2021; Jones et al., 2016; Kirby et al., 2013; Loane et al., 2007; Salinas-Torres et al., 2018). Further assessment could inform to what extent these two observations might be related. We also identified changes in the proportion of maternal age-specific births during the study period. The largest decline was observed in the proportion of births among women under 20 years and 20–24 years of age (approximately three-quarters of the programs) compared to women 25–29 years of age (<50% of the programs). Conversely, in most programs (>80%) the proportion of births among women ≥30 years of age increased. One important question is whether the observed increase in prevalence, especially among the youngest women, may be due to the changing population dynamics of women giving birth observed in this study. For example, in the United States, teen pregnancies have declined since 1991 (cCDC.gov/teen pregnancy), which may change maternal age specific prevalence. However, a more detailed investigation is required to assess each program’s specific changes in the maternal age-specific births in the population and the potential influence on prevalence over time. Generally, the increasing trends for gastroschisis appear to be primarily driven by the increasing occurrence among women under 20 years of age (the highest prevalence age group), relative to older maternal age groups. One might postulate that either a common exposure or exposures that induce a similar response (e.g., cell death at the umbilical region of the pars flaccida) among young women may explain this trend. Understanding environmental risk factors that are common in different populations and/or a maternal response periconceptionally or in early pregnancy that results in a gastroschisis is critical to reducing its occurrence, particularly among the youngest women. Improvements in our study methodology will be required to obtain measurable biomarkers either during the first trimester or as close to the first trimester as possible to assess modifiable exposures that are difficult to capture with maternal interviews.

### 4.1 | Strengths

We used strict inclusion criteria for case definition and excluded cases suggestive of limb–body wall complex, amniotic band sequence, or a ruptured omphalocele. Our study required each program to submit data for the entire surveillance period when gastroschisis was monitored in their country or region. This resulted in ~38 years of longitudinal data for analysis. Most programs in the study (18, or 67%) were population-based and monitored all pregnancy outcomes when legally permitted.

### 4.2 | Limitations

This study has limitations that may influence our findings. Though several surveillance systems collected data for the entire country (e.g., Costa Rica and Wales), the majority were regional. Some programs were hospital-based only (cases and the source population) making it difficult to generalize the study’s findings. Surveillance programs may vary in the process used to identify potential cases and prevalence estimates may increase over time as a program improves its ability to identify potential cases. In addition, the period for gastroschisis surveillance varied by program, which may make comparisons between programs problematic. Some programs were unable to provide maternal age for all cases and births in the population, which could under- or over-estimate prevalence. Differences in ascertainment may vary by program or over time within a program which may alter prevalence estimates for this study. Both population- and hospital-based surveillance programs may miss cases of gastroschisis, particularly if a pregnancy termination is permitted and occurred in a private clinic. Population-based studies suggest that the proportion of gastroschisis-related stillbirths is low, under 5% (Calzolari et al., 1995; Feldkamp et al., 2015; Sparks et al., 2017). In this study, stillbirths were higher in Latin America (5.9% of cases, range 2.9–33.3%) and North America (8.4% of cases, range 5.5–16.2%) and lowest in European^+^ (2.5% of cases, range 0.4–25%) suggesting that most of these programs were able to capture these cases. Pregnancy termination after prenatal diagnosis of fetal gastroschisis varies widely from 1.5% in Utah (Feldkamp et al., 2015), 14% in the Netherlands (Fleurke-Rozema et al., 2017) and 26.5% based on data from five registries in Italy (Calzolari et al., 1995). Though pregnancy termination practices vary by country and region, this study included gastroschisis cases from programs where termination is legal and monitored by surveillance programs. In this study, pregnancy terminations were much higher in European^+^ (31.6% of cases, range 0–54%) than North American (2.1% of cases, range 1.5%–4.9%) programs (see Table 1). Prenatal diagnosis of gastroschisis is most accurate at or after the 18–20 week diagnostic ultrasound, since the diagnosis of gastroschisis is distinct from other types of abdominal defects at this time, such as omphalocele and limb–body wall complex (Pakdaman et al., 2015). Distinguishing different abdominal defects by ultrasound in the first trimester can be challenging. Based on a study by Bogers et al. (2019) the presence of the normal physiologic hernia occurs between 35 and 70 days post-conception in 95% of women studied, making it difficult to distinguish between gastroschisis and omphalocele. In our study, gastroschisis case counts included both stillbirths and pregnancy terminations among those programs where terminations were permitted. We did not request information on race and ethnicity from programs since this is not useful to evaluate in this multinational project. As social constructs, the usefulness of race and ethnicity is complicated by individuals identifying as multiracial and often defined differently by country or region (Erayil et al., 2021).

### 4.3 | Summary

During the study period, gastroschisis prevalence increased in 61% of participating programs with an increase in all maternal age categories overall and by region. The highest prevalence occurred among the youngest women. With the prevalence of gastroschisis increasing in many areas of the world among those that currently monitor its occurrence, understanding the potential modifiable maternal and/or environmental exposures during the critical period of development is important to reverse this trend. We also observed a change in the proportion of population births by maternal age, shifting from younger to older women in many programs, which may result in a decreasing prevalence over time among the youngest women. Additional inquiry into this finding could help determine the contribution of changing dynamics of the birth population for each program and the relationship with gastroschisis prevalence. This study contributes important information on gastroschisis prevalence including recent time trends and differences by maternal age and region.

## Figures and Tables

**TABLE 1:** Characteristics of birth defect registries (27 birth defect programs representing 24 countries), International Clearinghouse for Birth Defects Surveillance and Research, 1980–2017.

Surveillance program by region	Coverage	Elective termination of pregnancy	Stillbirth criteria	Available years for gastroschisis prevalence	Available years for maternal age in gastroschisis cases and source population
*European*+					
Czech Republic	P, N	A, I	≥22 wks or >500 g	1980–2017	1994–2015
France: Paris	P, R	A, I	≥22 wks	1983–2017	1983–2017
France: REMERA	P, R	A, I	≥22 wks	1980–2017	1988–2017
Germany: Saxony-Anhalt	P, R	A, I	>500 g	1980–2017	1990–2017
Hungary	P, N	A, I	≥24 wks or ≥500 g	1982–2014	2005–2014^b^
Italy: Lombardy	P, R	A, I	≥23 wks	1999–2013	1999–2013
Italy: Tuscany RTDC	P, R	A, I	≥20 wks	1992–2017	1992–2017
Malta: MCAR	P, N	NA	≥22 wks	1999–2017	1999–2017
Northern Netherlands	P, R	A, I	≥24 wks	1981–2017	2000–2017
Slovak Republic	P, N	A, I	>500 g	1995–2016	2001–2016
Spain: ECEMC	H, R	A, NI	≥24 wks or >500 g	1980–2016	1980–2016
Wales: CARIS	P, R	A	≥24 wks	1998–2017	1998–2017
Iran TRoCA	H, R	A, R	≥20 wks or >500 g	2005–2017	NA
*Latin American*					
Argentina: RENAC	H, N	NA	>500 g	2010–2017	2010–2017
Chile-Maule: RRMC-SSM	H, R	NA	≥500 g	2002–2017	2002–2017
Colombia: Bogota	H, R	A^a^	>500 g	2003–2015	2003–2015^b^
Colombia: Cali	H, R	A^a^	>500 g	2011–2015	2011–2015^b^
Costa Rica: CREC	P, N	NA	>500 g	1996–2017	1996–2017
Mexico: Neuvo León	H, R	NA	Not included	2011–2015	2011–2015
Mexico: RYVEMCE	H, R	NA	≥20 wks or ≥500 g	1980–2017	1980–2017
South America: ECLAMC	H, R	NA	>500 g	1982–2017	1982–2017^b^
*North American*					
Canada: Alberta	P, S	A, I	≥20 wks or ≥500 g	1980–2016	1980–2016
Canada: CCASS	P, N	A, I	≥20 wks or ≥500 g	2004–2017	2004–2017
USA: Arkansas	P, S	A, I	≥20 wks	1993–2015	1993–2015
USA: Atlanta	P, R	A, I^c^	≥20 wks	1980–2016	1974–2016
USA: Texas	P, S	A, I	≥20 wks	1996–2016	1996–2016
USA: Utah	P, S	A, I	≥20 wks	1997–2017	1997–2017

*Note*: Coverage: P, population-based; H, hospital based; R, regional; N, national; S, statewide/provincewide. Terminations of Pregnancy: A, allowed by country’s legislation; NA, not allowed; I, included; NI, not included.

Abbreviations: CARIS, Congenital Anomaly Register and Information Services for Wales; CCASS, Canadian Congenital Anomalies Surveillance System; CREC, Costa Rican Birth Defects Register Center; ECLAMC, Latin American Collaborative Study of Congenital Malformations; ECEMC, Spanish Collaborative Study of Congenital Malformations; MCAR, Malta Congenital Anomalies Register; REMERA, Registre des Malformations en Rhône-Alpes; RENAC, National Network of Congenital Anomalies of Argentina; RRMC-SSM, Registro de malformaciones congénitas del Servicio de Salud Maule; RTDC, Registro Toscano Difetti Congeniti; RYVEMCE, Registration and Epidemiologic Surveillance of Congenital Malformations; TRoCA, Tabriz Registry of Congenital Anomalies.

a Permitted since 2006.

b Population denominator based on hospital births only.

c Ascertainment started in 1994.

**TABLE 2:** Gastroschisis prevalence overall and by decade, among 27 programs representing 24 countries, International Clearinghouse for Birth Defects Surveillance and Research, 1980–2017.

Country—registry	Number of cases	Total births (live and stillbirth)	Prevalence (95% Cl) per 10,000 births	1980–1989	1990–1999	2000–2009	2010–2017	*P* value	Change
*European+*	*2915*	*19,527,489*	*1.49 (1.44, 1.55)*						
Czech Republic	1106	4,373,228	2.53 (2.38, 2.68)	1.32(1.13, 1.52)	2.71 (2.40, 3.02)	2.93 (2.60, 3.27)	3.71 (3.31, 4.11)	<.0001	⬆
France: Paris	146	898,856	1.62 (1.36, 1.89)	1.02 (0.55, 1.49)	1.92 (1.38, 2.47)	1.76 (1.26, 2.27)	1.60(1.06, 2.15)	0.5491	
France: REMERA	457	3,232,057	1.41 (1.28, 1.54)	0.87 (0.67, 1.07)	1.33(1.11, 1.56)	1.72 (1.45, 2.00)	2.02 (1.61, 2.42)	<.0001	⬆
Germany: Saxony-Anhalt	162	579,832	2.79 (2.36, 3.22)	0.76 (0.35, 1.17)	3.09 (1.96, 4.21)	3.76 (2.85, 4.66)	3.89 (2.85, 4.93)	<.0001	⬆
Hungary	254	3,639,646	0.70 (0.61, 0.78)	0.50 (0.36, 0.63)	0.61 (0.46, 0.75)	0.94 (0.75, 1.13)	0.83 (0.58, 1.08)	<.0001	⬆
Italy: Lombardy	36	225,569	1.60(1.07, 2.12)		1.20 (0.03, 6.67)	1.55 (0.89, 2.21)	1.71 (0.82, 2.61)	0.7497	
Italy: Tuscany RTDC	55	715,172	0.77 (0.57, 0.97)		0.45 (0.16, 0.74)	0.76 (0.44, 1.08)	1.06 (0.64, 1.48)	0.0186	⬆
Malta: MCAR	9	79,153	1.14 (0.39, 1.88)		2.28 (0.06, 12.71)	0.99 (0.27, 2.54)	1.16 (0.32, 2.97)	0.7509	
Northern Netherlands	71	608,827	1.17 (0.89, 1.44)	0.97 (0.34, 1.60)	0.67 (0.30, 1.03)	1.10 (0.63, 1.57)	2.16 (1.36, 2.96)	0.0002	⬆
Slovak Republic	128	1,242,778	1.03 (0.85, 1.21)		0.78 (0.46, 1.10)	1.06 (0.79, 1.34)	1.17 (0.84, 1.51)	0.1889	
Spain: ECEMC	143	3,079,418	0.46 (0.39, 0.54)	0.50 (0.32, 0.68)	0.40 (0.27, 0.53)	0.52 (0.38, 0.66)	0.42 (0.25, 0.60)	0.7555	
Wales: CARIS	344	668,195	5.15 (4.60, 5.69)		5.31 (3.55, 7.07)	5.97 (5.13, 6.80)	4.13 (3.37, 4.89)	0.0306	⬇
Iran TRoCA	4	184,758	0.22 (0.06, 0.55)				0.52 (0.14, 1.34)^a^	nc	
*Latin American*	*4321*	*11,359,857*	*3.80 (3.69, 3.92)*						
Argentina: RENAC	1531	1,934,725	7.91 (7.52, 8.31)				7.91 (7.52, 8.31)	nc	
Chile-Maule: RRMC-SSM	53	211,743	2.50(1.83, 3.18)			1.88 (1.06, 2.71)	3.13 (2.06, 4.20)	0.0052	⬆
Colombia: Bogota	105	364,224	2.88 (2.33, 3.43)			2.78 (1.62, 3.95)	2.91 (2.28, 3.54)	0.9192	
Colombia: Cali	6	35,189	1.71 (0.34, 3.07)				1.71 (0.34, 3.07)	nc	
Costa Rica: CREC	341	1,631,106	2.09 (1.87, 2.31)		1.02 (0.67, 1.37)	1.70 (1.40, 2.00)	3.19 (2.73, 3.65)	<.0001	⬆
Mexico: Nuevo León	71	445,313	1.59 (1.22, 1.97)				1.59 (1.22, 1.97)	nc	
Mexico: RYVEMCE	343	1,179,029	2.91 (2.60, 3.22)	0.84 (0.54, 1.14)	2.42 (1.99, 2.86)	5.41 (4.47, 6.35)	7.31 (5.54, 9.09)	<.0001	⬆
South America: ECLAMC	1871	5,558,528	3.37 (3.21, 3.52)	0.57 (0.45, 0.69)	1.79 (1.59, 1.99)	5.08 (4.75, 5.41)	9.41 (8.64, 10.17)	<.0001	⬆
*North American*	*7125*	*16,498,897*	*4.32 (4.22, 4.42)*						
Canada: Alberta	470	1,632,552	2.88(2.62, 3.14)	1.39 (1.04, 1.75)	2.07 (1.62, 2.52)	4.36 (3.74, 4.99)	3.74 (3.12, 4.36)	<.0001	⬆
Canada: CCASS^b^	1484	3,832,840	3.87 (3.67, 4.07)			3.85 (3.54, 4.15)	3.89 (3.63, 4.15)	0.4185	
USA: Arkansas	471	867,879	5.43 (4.94, 5.92)		4.59 (3.75, 5.43)	5.09 (4.38, 5.79)	6.93 (5.85, 8.01)	<.0001	⬆
USA: Atlanta	424	1,526,873	2.78 (2.51, 3.04)	2.02 (1.52, 2.52)	2.15(1.70, 2.60)	3.67 (3.15, 4.18)	2.85 (2.22, 3.48)	0.0002	⬆
USA: Texas	3805	7,577,857	5.02(4.86, 5.18)		3.74(3.35,4.13)	4.95 (4.72, 5.17)	5.57 (5.29, 5.85)	<.0001	⬆
USA: Utah	471	1,060,896	4.44 (4.04, 4.84)		4.22 (3.13, 5.32)	4.78 (4.18, 5.37)	4.08 (3.46, 4.70)	0.7627	
*Total*	*14,020*	*45,755,137*	*3.06(3.01, 3.11)*						

a Iran did not report any cases occurring during the surveillance period 2005–2009 but submitted denominator data.

b CCASS excludes data from Quebec and Alberta.

Abbreviations: CARIS, Congenital Anomaly Register and Information Services for Wales; CCASS, Canadian Congenital Anomalies Surveillance System; CI, confidence interval; CREC, Costa Rican Birth Defects Register Center; ECLAMC, Latin American Collaborative Study of Congenital Malformations; ECEMC, Spanish Collaborative Study of Congenital Malformations; MCAR, Malta Congenital Anomalies Register; nc, not calculated; REMERA, Registre des Malformations en Rhône-Alpes; RENAC, National Network of Congenital Anomalies of Argentina; RRMC-SSM, Registro de malformaciones congénitas del Servicio de Salud Maule; RTDC, Registro Toscano Difetti Congeniti; RYVEMCE, Registration and Epidemiologic Surveillance of Congenital Malformations; TRoCA, Tabriz Registry of Congenital Anomalies.

**TABLE 3:** Gastroschisis prevalence by maternal age among 22 birth defect programs representing 15 countries, International Clearinghouse for Birth Defects Surveillance and Research, 1980–2017.

			Prevalence (95% confidence interval) per 10,000 births			
			
Country—Registry^a^	Number of cases^b^	Total births^b^,^c^ (live and stillbirth)	Maternal age <20	Maternal age 20–24	Maternal age 25–29	Maternal age 30+
*European*	2102	12,064,233	8.04 (7.19, 8.88)	3.78 (3.51, 4.05)	1.5 (1.38, 1.62)	0.75 (0.68, 0.82)
Czech Republic	682	2,258,137	8.90 (7.12, 10.69)	5.17 (4.55, 5.79)	2.65 (2.29, 3.01)	1.32 (1.07, 1.56)
France: Paris	146	873,480	12.75 (5.82, 19.67)	4.04 (2.68, 5.39)	1.85 (1.31, 2.40)	1.01 (0.74, 1.28)
France: REMERA	392	2,560,141	8.95 (6.15, 11.76)	2.72 (2.20, 3.23)	1.40 (1.16, 1.64)	0.98 (0.80, 1.16)
Germany: Saxony-Anhalt	144	403,329	9.82 (5.72, 13.93)	6.96 (5.26, 8.67)	2.45 (1.63, 3.27)	1.60 (0.96, 2.24)
Italy: Lombardy	36	222,072	14.88 (4.06, 38.06)	4.82 (1.67, 7.97)	1.33 (0.34, 2.31)	1.08 (0.55, 1.61)
Italy: Tuscany RTDC	53	709,593	5.61 (0.69, 10.53)	2.04 (0.93, 3.14)	0.79 (0.38, 1.21)	0.46 (0.26, 0.65)
Malta: MCAR	9	78,743	2.43 (0.06, 13.56)	2.42 (0.50, 7.06)	0.76 (0.09, 2.73)	0.84 (0.17, 2.45)
Northern Netherlands	49	320,584	8.97 (2.45, 22.96)	4.58 (2.26, 6.89)	1.29 (0.59, 1.99)	0.93 (0.49, 1.37)
Slovak Republic	94	890,543	2.05 (0.94, 3.16)	2.54 (1.82, 3.25)	0.67 (0.38, 0.96)	0.38 (0.18, 0.59)
Spain: ECEMC	153	3,079,414	3.66 (2.54, 4.78)	1.27 (0.93, 1.61)	0.37 (0.24, 0.50)	0.15 (0.09, 0.21)
Wales: CARIS	344	668,197	20.61 (16.76, 24.46)	9.17 (7.61, 10.74)	3.58 (2.73, 4.43)	1.21 (0.80, 1.62)
*Latin American*	4210	5,491,318	10.31 (9.70, 10.92)	4.88 (4.54, 5.23)	2.23 (1.97, 2.49)	1.11 (0.94, 1.28)
Argentina: RENAC	1516	1,933,202	19.5 (18.08, 20.92)	9.27 (8.47, 10.08)	4.05 (3.45, 4.65)	1.82 (1.47, 2.17)
Chile-Maule: RRMC-SSM	53	216,377	8.12 (5.00, 11.24)	3.67 (2.02, 5.33)	0.95 (0.12, 1.78)	0.38 (0.08, 1.10)
Costa Rica: CREC	336	1,613,847	5.04 (4.25, 5.83)	3.67 (2.02, 5.33)	4.05 (3.45, 4.65)	1.89 (1.47, 2.31)
Mexico: Nuevo León	71	440,538	5.18 (3.49, 6.87)	1.63 (0.92, 2.35)	1.00 (0.41, 1.59)	0.29 (0.08, 0.74)
Mexico: RYVEMCE	333	1,166,098	5.42 (4.55, 6.30)	3.05 (2.50, 3.59)	1.51 (1.04, 1.98)	1.03 (0.62, 1.45)
*North American*	7135	16,489,703	15.62 (15.00, 16.24)	7.53 (7.25, 7.80)	2.58 (2.44, 2.73)	0.92 (0.85, 0.99)
Canada: Alberta	470	1,630,687	12.86 (10.64, 15.08)	5.55 (4.76, 6.34)	1.95 (1.58, 2.32)	0.70 (0.49, 0.90)
Canada: CCASS^d^	1482	3,845,792	26.21 (23.6, 28.82)	10.42 (9.57, 11.27)	3.06 (2.73, 3.38)	0.87 (0.74, 1.00)
USA: Arkansas	483	863,986	12.81 (10.89, 14.73)	7.51 (6.51, 8.52)	2.71 (2.05, 3.37)	1.62 (1.08, 2.17)
USA: Atlanta	424	1,519,099	8.73 (7.21, 10.25)	4.85 (4.11, 5.59)	1.89 (1.47, 2.31)	0.86 (0.63, 1.09)
USA: Texas	3805	7,574,908	15.31 (14.53, 16.09)	7.62 (7.24, 8.00)	2.59 (2.37, 2.81)	0.97 (0.85, 1.09)
USA: Utah	471	1,055,231	21.47 (17.98, 24.96)	6.80 (5.85, 7.77)	2.77 (2.23, 3.32)	0.98 (0.65, 1.31)
Total	13,447	34,045,254	12.70 (12.30, 13.10)	5.96 (5.79, 6.14)	2.12 (2.02, 2.21)	0.87 (0.82, 0.92)

Abbreviations: CARIS, Congenital Anomaly Register and Information Services for Wales; CCASS, Canadian Congenital Anomalies Surveillance System; CREC, Costa Rican Birth Defects Register Center; ECLAMC, Latin American Collaborative Study of Congenital Malformations; ECEMC, Spanish Collaborative Study of Congenital Malformations; MCAR, Malta Congenital Anomalies Register; REMERA, Registre des Malformations en Rhône-Alpes; RENAC, National Network of Congenital Anomalies of Argentina; RRMC-SSM, Registro de malformaciones congénitas del Servicio de Salud Maule; RTDC, Registro Toscano Difetti Congeniti; RYVEMCE, Registration and Epidemiologic Surveillance of Congenital Malformations; TRoCA, Tabriz Registry of Congenital Anomalies.

a Excluded Hungary, Colombia Bogota, Colombia Cali and South America ECLAMC—denominator data not population-based (based on hospital births).

b Number of cases and denominator data may be different from the prevalence numbers if maternal age was unknown, or if data were not available for all birth years (see Table 1).

c Maternal age may not have been available for all stillbirths.

d Canada’s National program excluded Quebec and Alberta.

## Data Availability

Data sharing is not applicable to this article as no new data were created or analyzed in this study.
